# Supplementary material for: Alterations of gut microbiota contribute to the progression of unruptured intracranial aneurysms
Source: Nat Commun. 2020 Jun 25;11:3218. doi: 10.1038/s41467-020-16990-3 (PMC7316982; doi:10.1038/s41467-020-16990-3)
Supplement: Supplementary file 1 — Supplementary Information [file 41467_2020_16990_MOESM1_ESM.pdf]

Alterations of gut microbiota contribute to the progression of unruptured intracranial aneurysms

Li et al.

SUPPLEMENTAL MATERIAL

Supplementary Figures

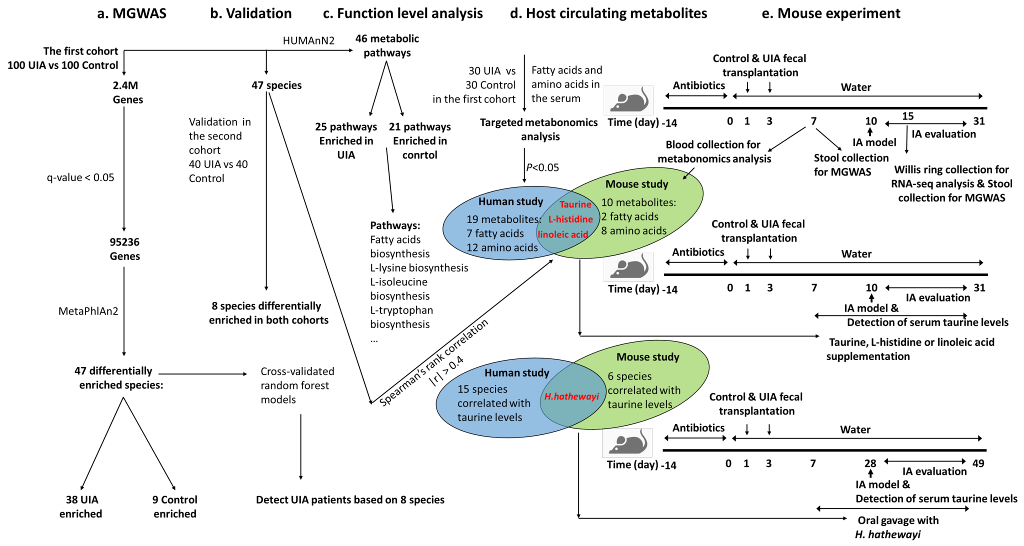

Supplementary Figure 1. Overview of workflow for the study design (a) Metagenomics data were aligned to the integrated gene catalog and MetaPhlAn2 (metagenomic phylogenetic analysis) was run to identify the taxonomic abundances at the species level. (b) Validation of the enrichment of 47 UIA-associated species in the second cohort. (c) The species-level functional profiling in UIA were identified using HUMAnN2 (the HMP Unified Metabolic Analysis Network 2) pipeline. (d) The difference of host circulating metabolites between control and UIA samples were validated by the targeted metabonomics analysis, which were further associated with the differentially abundant species. (e) Experimental design for fecal transplantation, taurine supplementation and *Hungatella hathewayi* gavage in mouse study.

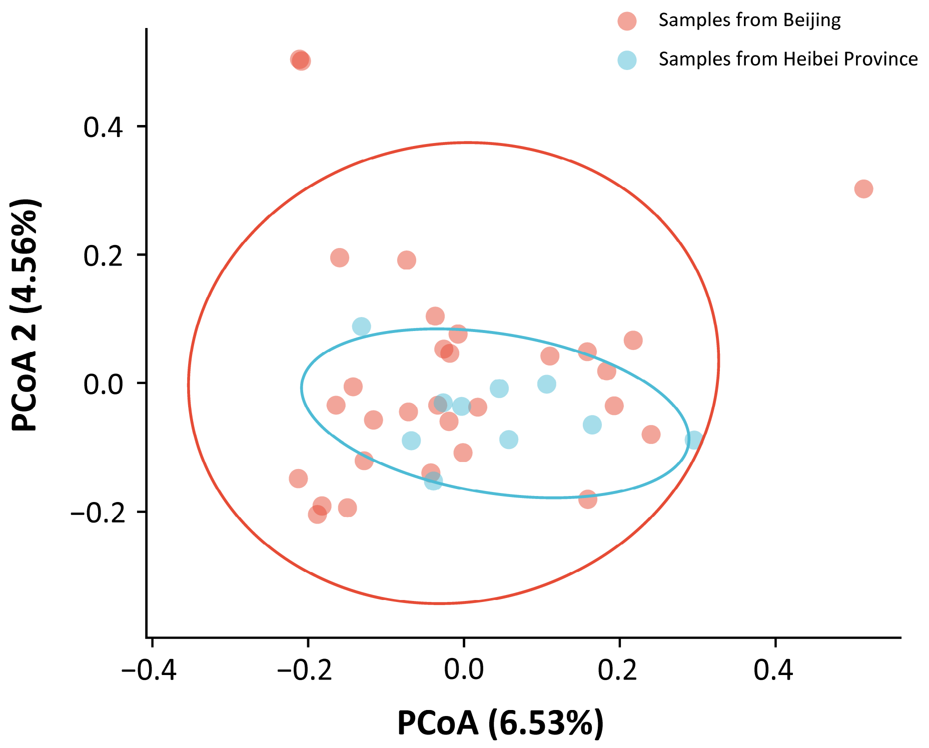

Supplementary Figure 2. Principal coordinate analysis (PCoA) for the control samples collected from different areas in the second cohort. Genera with the largest weights on each principal component are shown. n = 10 for samples collected from Cangzhou Central Hospital (Heibei Province), n = 30 for samples collected from Tsinghua University Hospital (Beiing).

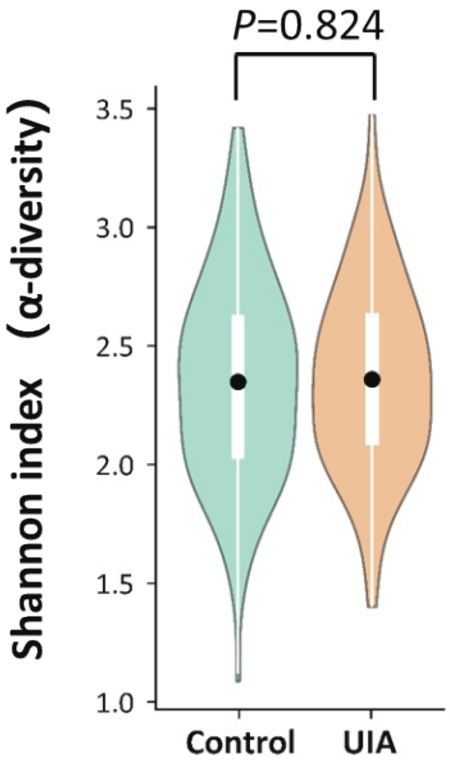

Supplementary Figure 3. Comparison of the α-diversity (as assessed by the Shannon index) at the species level between UIA patients and Controls. Interquartile ranges (IQRs; thick bars), medians (open dots on the bars), the lowest and highest values within 1.5 times IQR from the first and third quartiles (lines above and below the bars). n = 100. The two-tailed Wilcoxon rank-sum test. Source data are provided as a Source Data file.

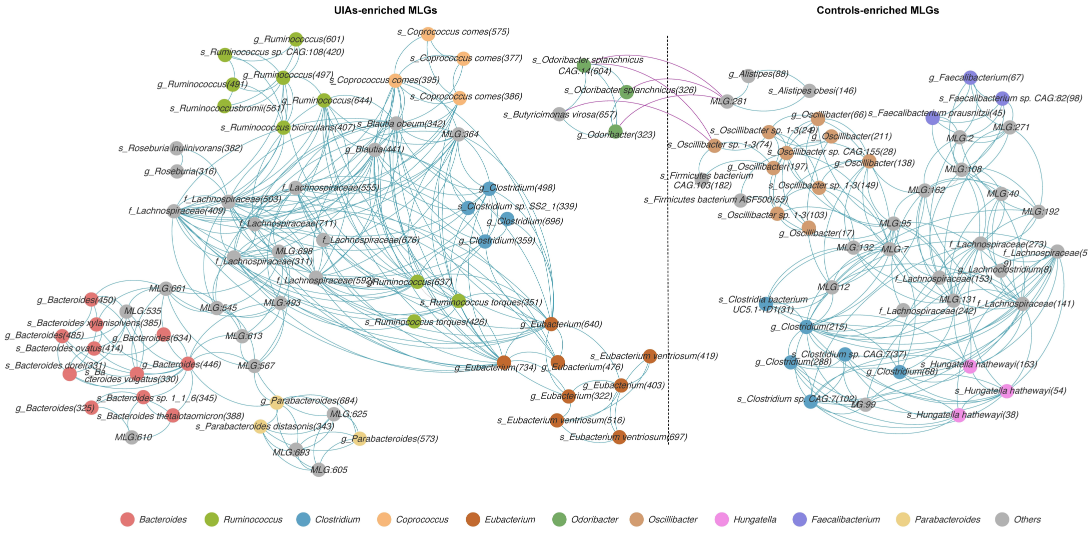

Supplementary Figure 4. SparCC (Sparse Correlations for Compositional data) correlation network of MLGs differentially enriched in individuals with and without UIAs. *Left*, network in UIA patients; *right*, network in controls. Nodes correspond to MLGs and connecting edges indicate correlations between them. Blue edges, correlation coefficient > 0.5, Benjamin-Hochberg corrected $P < 0.01$; Magenta edges, correlation coefficient < −0.5, Benjamin-Hochberg corrected $P < 0.01$. The numbers in parentheses next to each species name represent unique MLG identifiers.

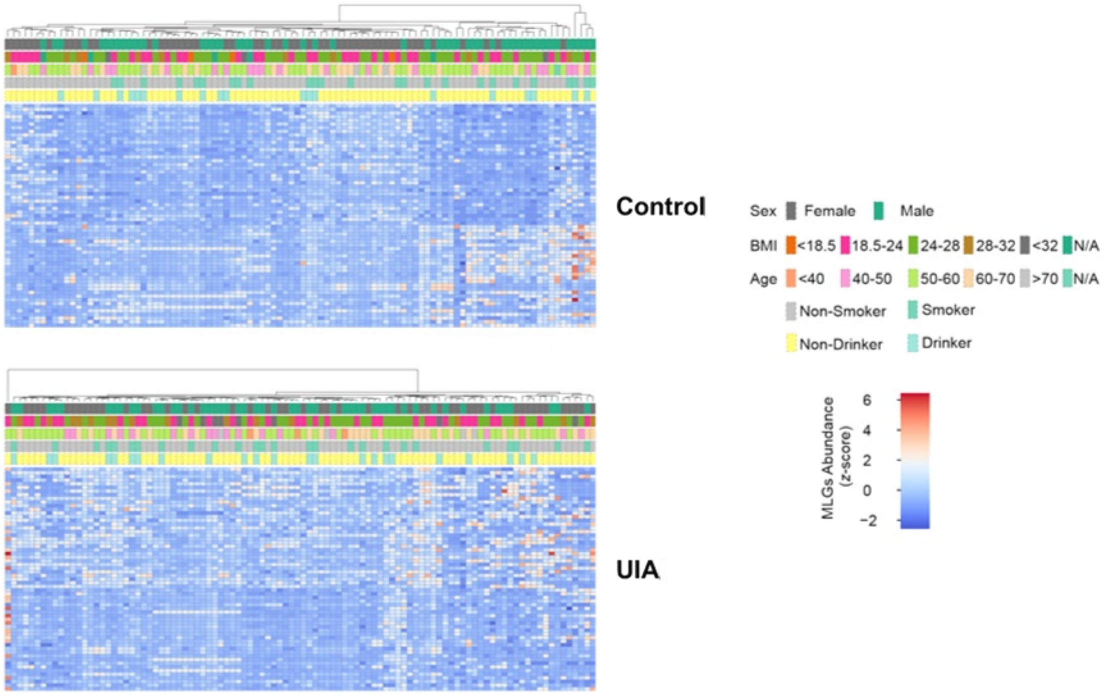

Supplementary Figure 5. Gut microbial abundances of MLGs enriched in groups are not correlated with age, BMI, sex, smoking, or alcohol drinking. The relative abundances of MLGs in controls ($n = 100$) and UIAs ($n = 100$) are shown. The information on age, BMI, sex, smoking, and alcohol drinking of each participant are included in the heat map.

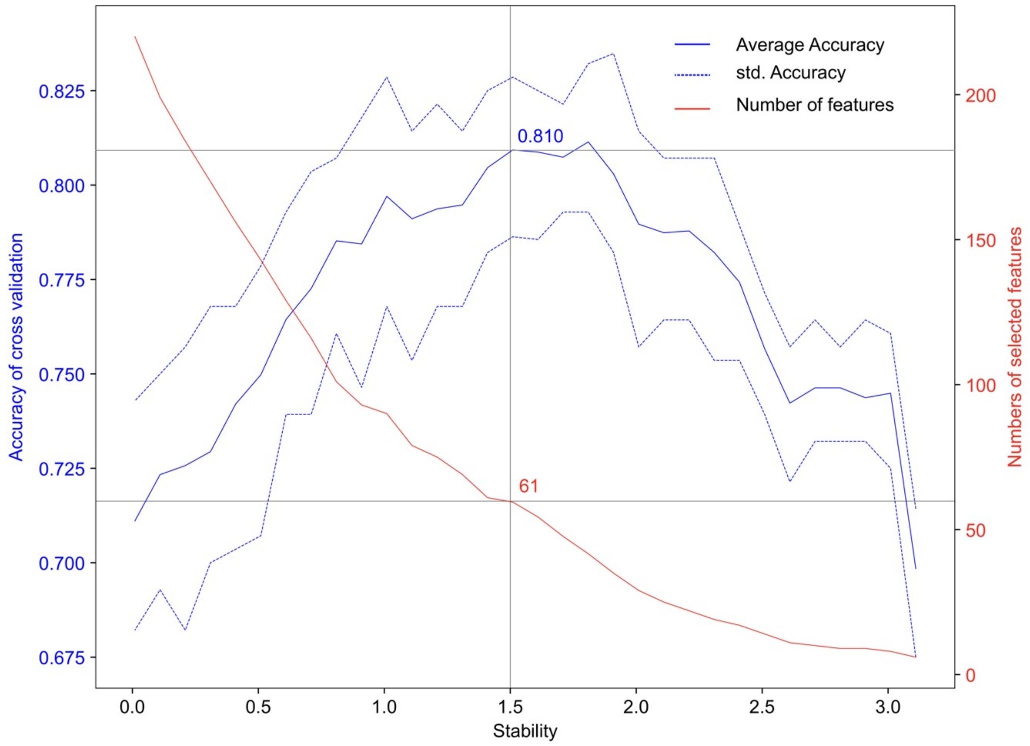

Supplementary Figure 6. Feature selection of distinct UIA-associated MLGs in the gut microbiome. The multivariate statistical analysis PLS-DA was performed to discriminate UIA samples from controls. The number of MLGs responsible for the differences in the microbiome profile scans of UIA patients and controls were based on the Monte Carlo cross-validation framework. The accuracy of cross-validation reached 81%, indicating that these 61 MLGs represent distinct UIA-associated features of the gut microbiome.

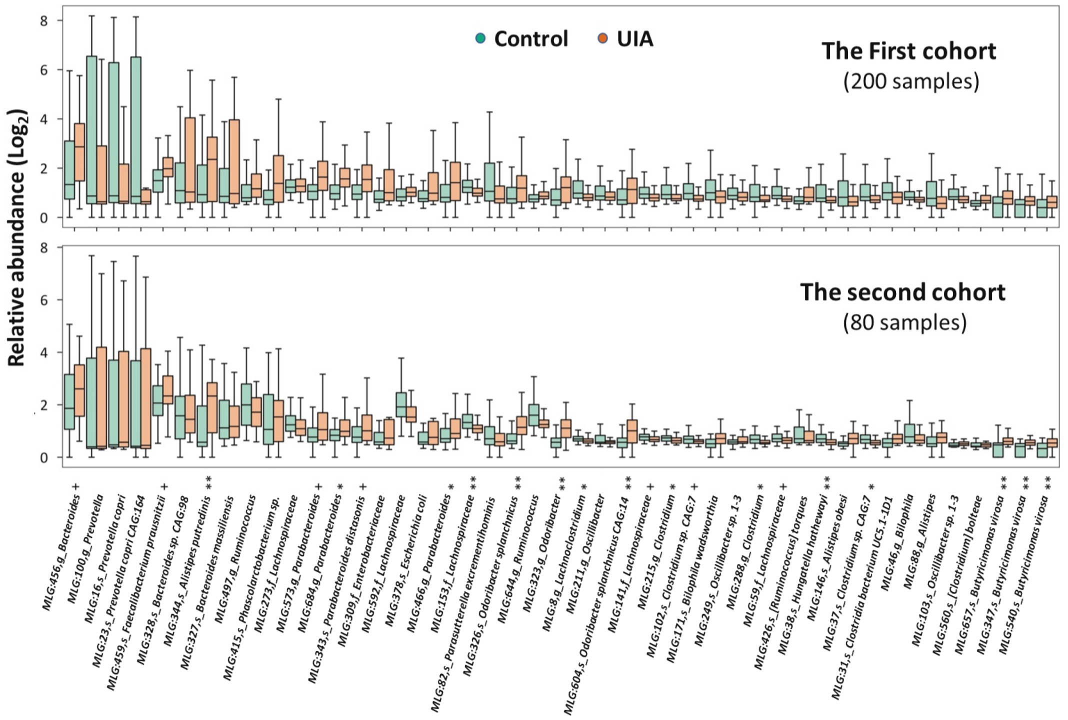

Supplementary Figure 7. Alterations of gut microbial MLGs in UIA patients. Turquoise and orange, abundance of control- and UIA-enriched MLGs; ^{+}P <0.1, $^{*}P$ <0.05, $^{**}P$ <0.01 for both the first (200 samples) and second cohorts (80 samples) (two-tailed Wilcoxon rank-sum test). In all box plots, boxes represent the interquartile ranges (IQRs) between the first and third quartiles, and the line inside the box represents the median; whiskers represent the lowest or highest values within 1.5 × IQR from the first or third quartiles. Source data are provided as a Source Data file.

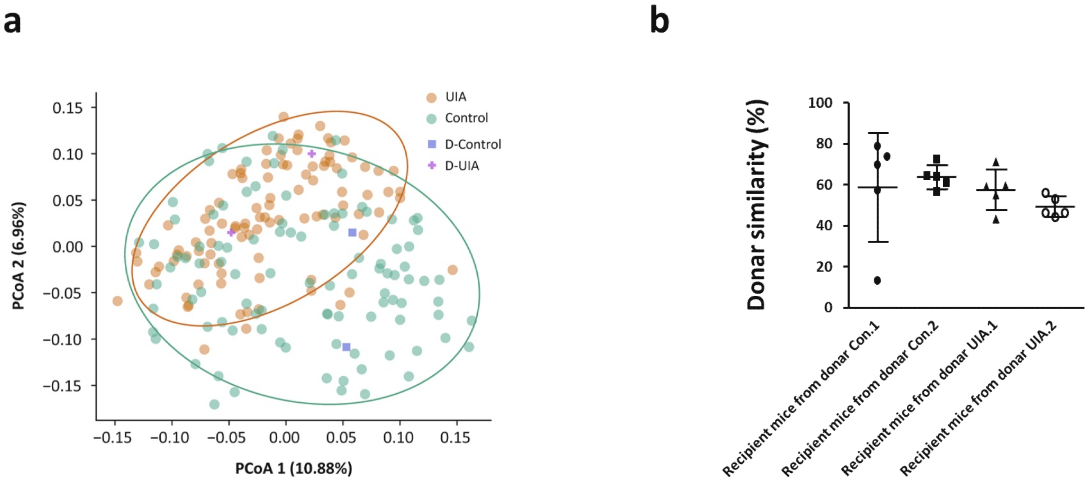

Supplementary Figure 8. Stable engraftment of human microbiota into mice after fecal transplantation. (a) Species with the largest weights on each principal component are shown. Turquoise and orange represent control donor- and UIA donor-enriched species classifications, respectively. (b) Donor similarity refers to the percent of the community attributable to the donor community, as determined by SourceTracker. Data are the mean ± SD. n = 5. Source data are provided as a Source Data file.

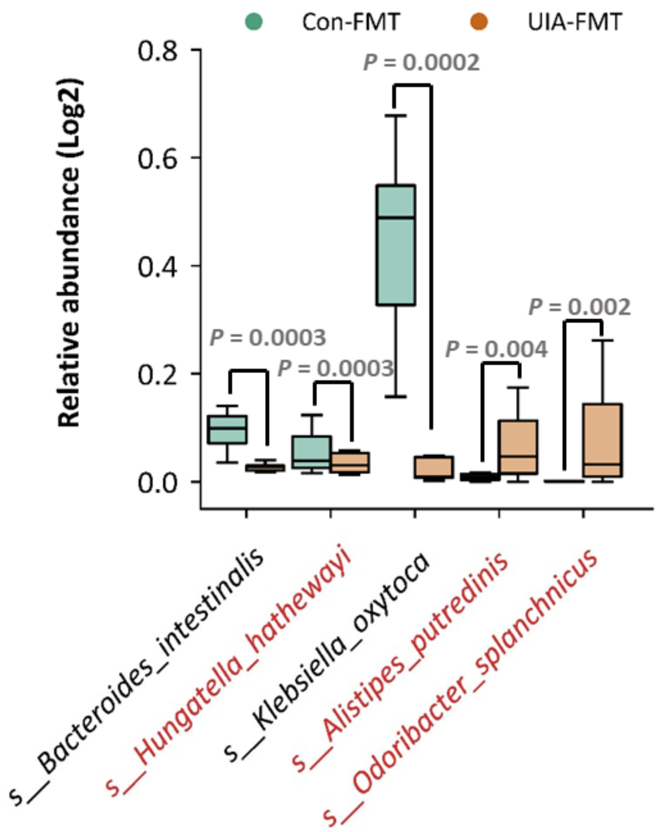

Supplementary Figure 9. Alterations of gut microbial species in mice after fecal transplantation. Turquoise and orange, abundance of Control- and UIA-enriched species. Crimson color indicates gut microbial species that were also differentially abundant in both human cohorts. n = 10. Two-tailed Wilcoxon rank-sum test. In all box plots, boxes represent the interquartile ranges (IQRs) between the first and third quartiles, and the line inside the box represents the median; whiskers represent the lowest or highest values within 1.5 × IQR from the first or third quartiles.

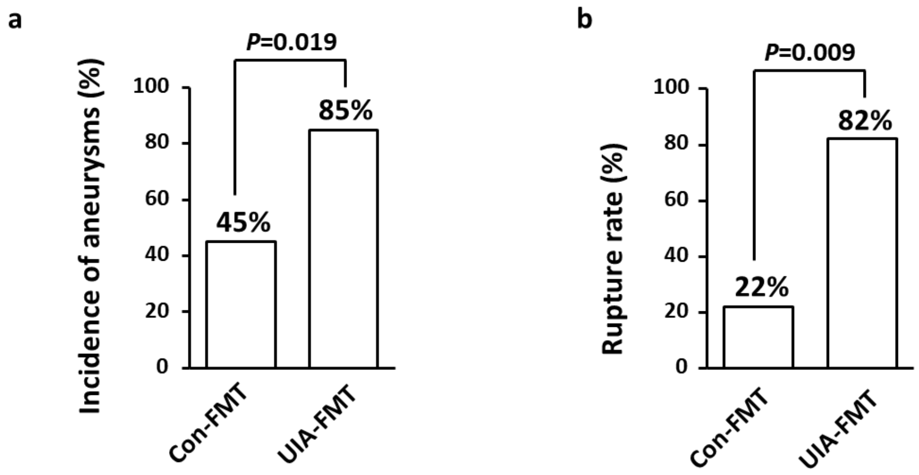

Supplementary Figure 10. Effects of fecal transplantation on the overall incidence and the rupture rate of intracranial aneurysms in mice. (a) Incidence of aneurysms at 21 days after aneurysm induction (n = 20). (b) Rupture rate (number of mice with ruptured aneurysms/number of mice with any aneurysms) (n = 17 for the Con-FMT group; n = 9 for the UIA-FMT group). Two-sided fisher's exact test; FMT, fecal microbiota transplantation.

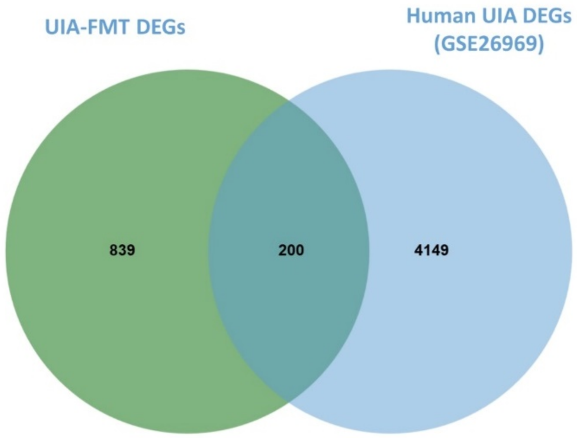

Supplementary Figure 11. The intersection between UIA-FMT-associated differentially-expressed genes (DEGs) and human UIA DEGs. Venn diagram shows the number of DEGs. The numbers in the teal circle represent DEGs induced by UIA gut microbiota after aneurysm induction. The numbers in the blue circle represent the DEGs in another human transcriptome-wide characterization associated with UIA (GSE26969). Source data are provided as a Source Data file.

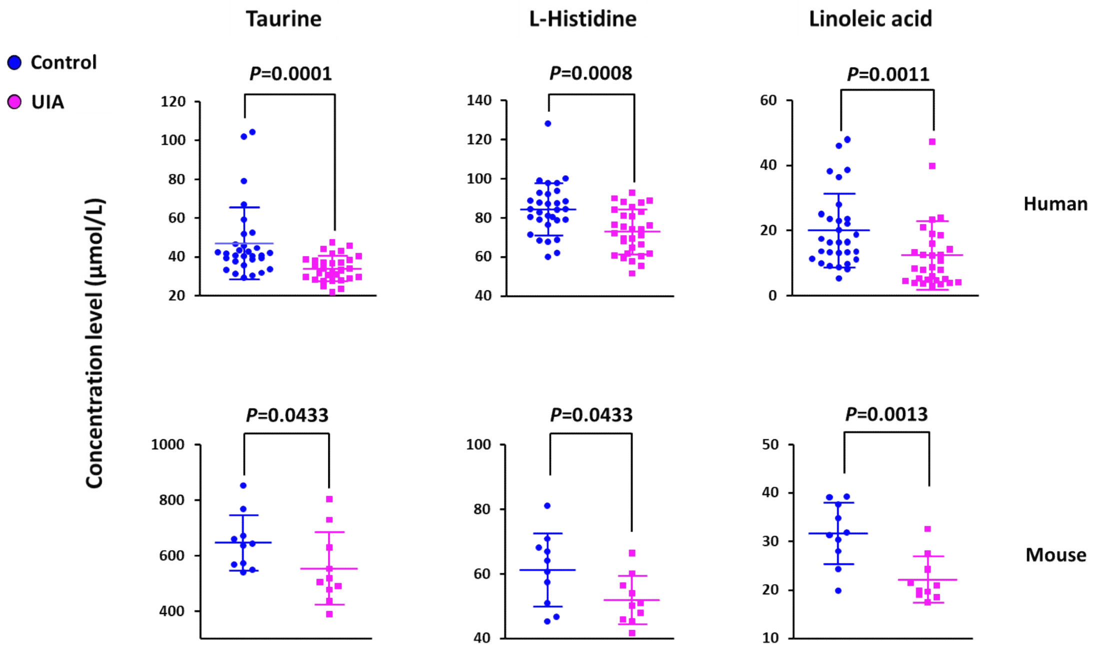

Supplementary Figure 12. Overlapping altered circulating metabolites in human patients and mice after fecal transplantation. The serum concentrations of taurine, L-histidine and linoleic acid were measured by targeted metabolomics profiling. In all scatterplots, data are the mean±SD. n=30 for human study; n=10 for mouse study. Student's unpaired two-tailed t-test or Mann-Whitney U test with the exact method. Source data are provided as a Source Data file.

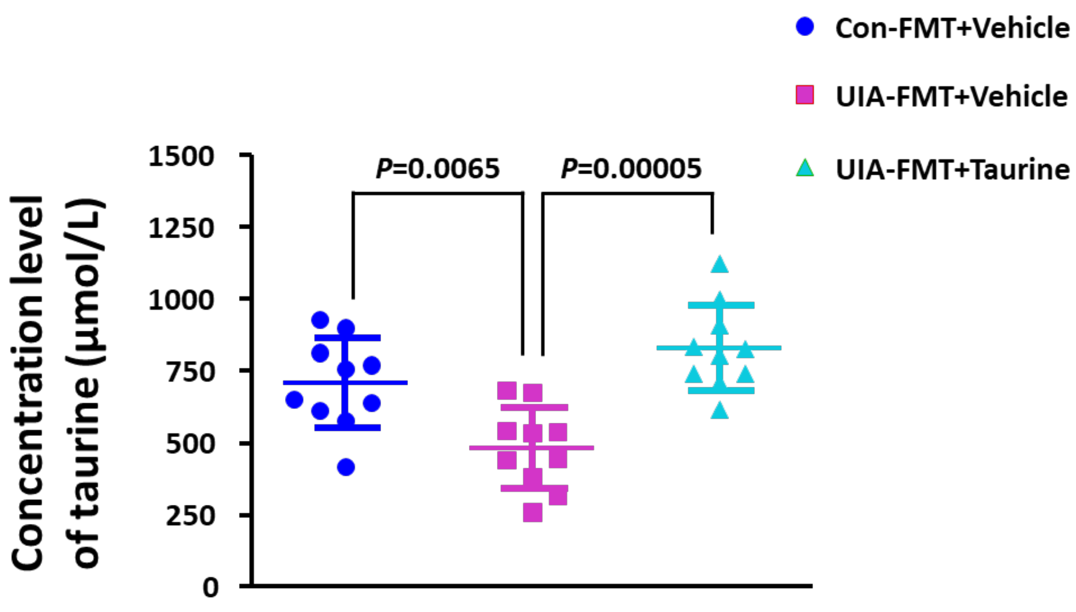

Supplementary Figure 13. Taurine supplementation normalized the serum levels of taurine after fecal transplantation in mice. The serum concentrations of taurine were measured by UHPLC-MS/MS analyses. Data are the mean±SD. n = 10; one-way ANOVA with Bonferroni post hoc test. Source data are provided as a Source Data file.

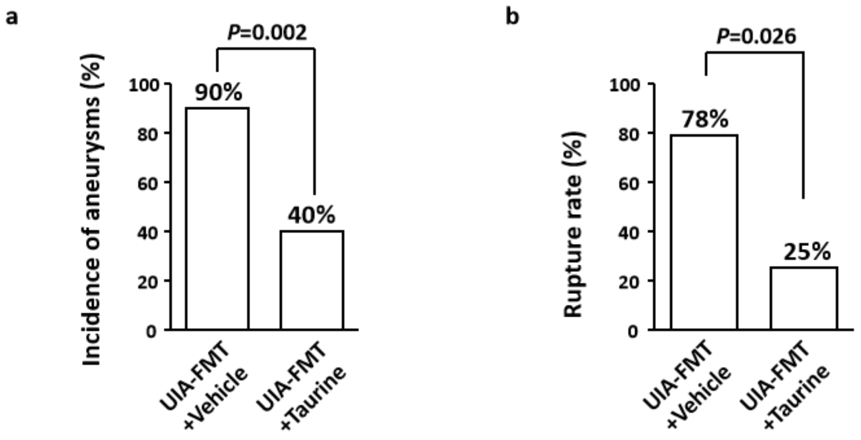

Supplementary Figure 14. Effects of taurine on the overall incidence and rupture rate of intracranial aneurysms after fecal transplantation in mice. (a) Incidence of aneurysms at 21 days after aneurysm induction (n = 20). (b) Rupture rate (number of mice with ruptured aneurysms/number of mice with any aneurysms) (n = 18 for the UIA-FMT+Vehicle group; n = 8 for the UIA-FMT+Taurine group). Two-sided fisher’s exact test; FMT, fecal microbiota transplantation.

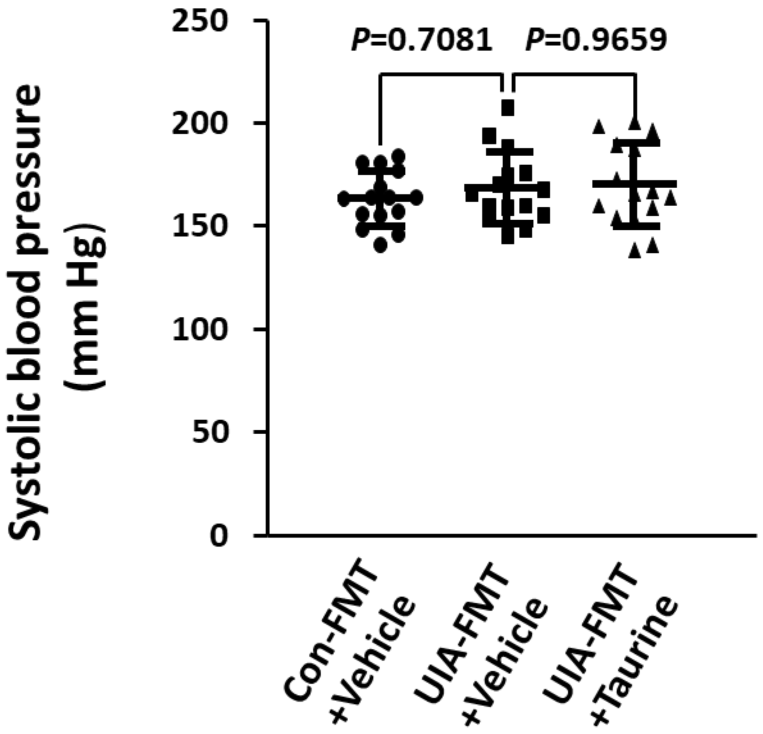

Supplementary Figure 15. Systolic blood pressure of mice subjected to fecal microbiota transplantation and aneurysm induction. Systolic blood pressure in each group on day 5 after aneurysm induction (n = 15). Data are presented as the mean ± SD. One-way ANOVA with the Bonferroni post hoc test. Source data are provided as a Source Data file.

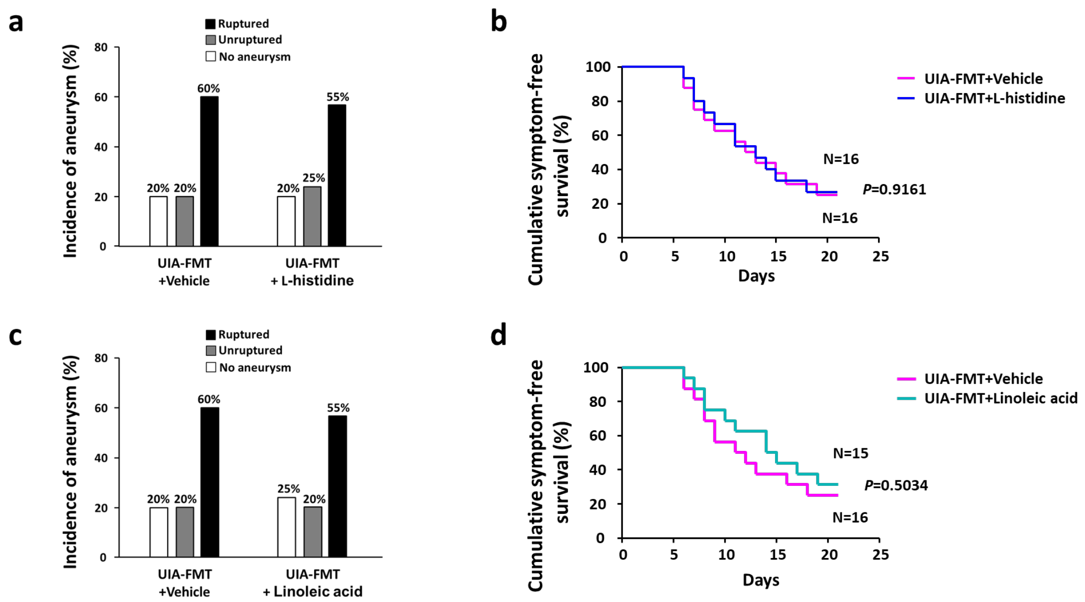

Supplementary Figure 16. Effects of L-histidine and linoleic acid on the formation and rupture of intracranial aneurysms in mice. (a) Incidence of unruptured and ruptured aneurysms after fecal microbiota transplantation (FMT), L-histidine supplementation and aneurysm induction in mice (n = 20). (b) Cumulative symptom-free curves for mice with aneurysms to show the time course of symptom onset after L-histidine supplementation (n = 16; log-rank (Mantel-Cox) test). (c) Incidence of unruptured and ruptured aneurysms after FMT, linoleic acid supplementation and aneurysm induction in mice (n = 20). (d) Cumulative symptom-free curves for mice with aneurysms to show the time course of symptom onset after linoleic acid supplementation (n = 15-16; log-rank (Mantel-Cox) test).

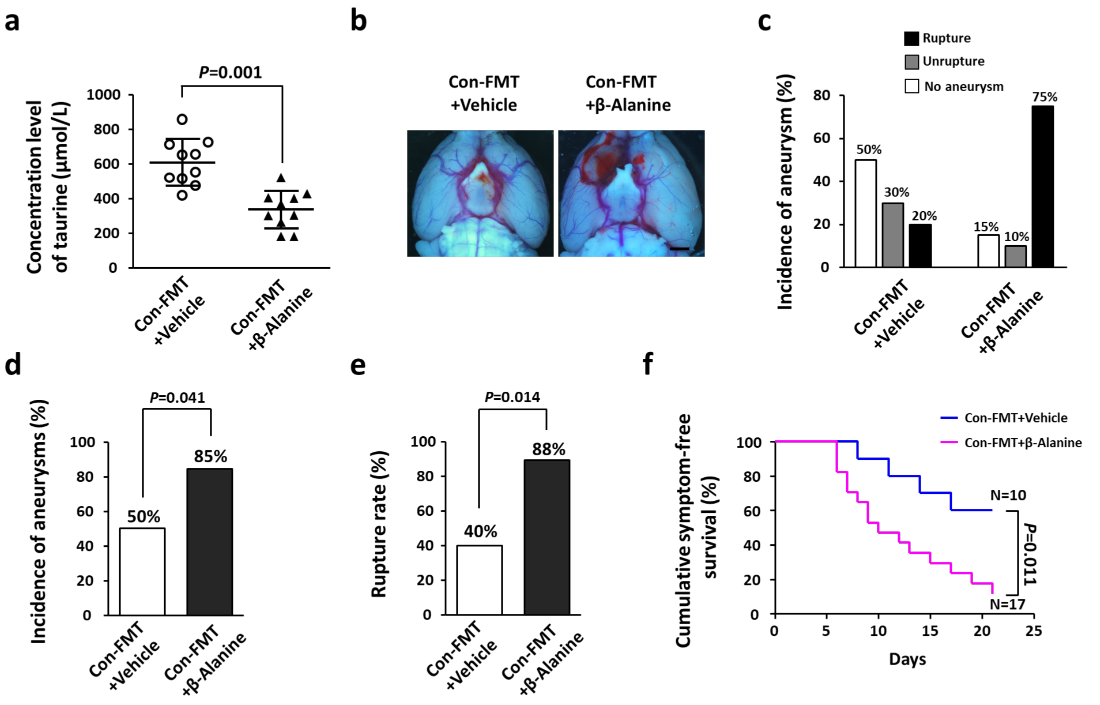

Supplementary Figure 17. Diminished level of circulating taurine increases the formation and rupture of intracranial aneurysms in mice. (a) Serum concentrations of taurine after β-alanine or Vehicle treatment measured by UHPLC-MS/MS (n = 10; Student's unpaired two-tailed t-test).Data are presented as the mean ± SD. (b) Representative images of intracranial aneurysms induced with angiotensin II and elastase in each group (scale bar, 1 mm). (c) Incidence of unruptured and ruptured aneurysms 21 days after aneurysm induction (n = 20). (d) Incidence of aneurysms at 21 days after aneurysm induction (n = 20). (e) Rupture rate (number of mice with ruptured aneurysms/number of mice with any aneurysms) (n = 10 for the Con-FMT+Vehicle group; n = 17 for the Con-FMT+β-alanine group). Two-sided fisher's exact test; FMT, fecal microbiota transplantation. (f) Cumulative symptom-free curves for mice with aneurysms to show the time course of symptom onset (n = 10–17; log-rank (Mantel–Cox) test). Source data are provided as a Source Data file.

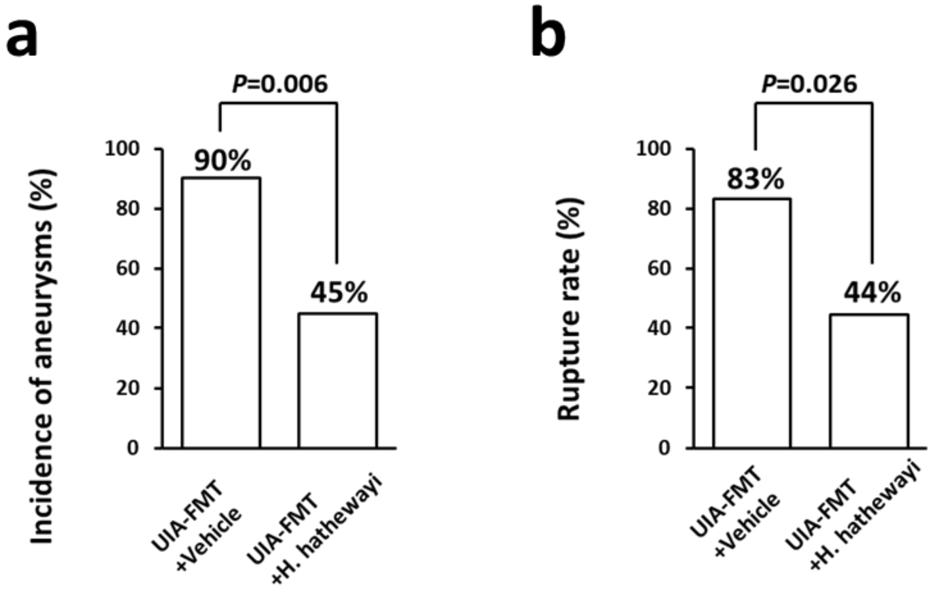

Supplementary Figure 18. Effects of *H. hathewayi* on the overall incidence and rupture rate of intracranial aneurysms after fecal transplantation in mice. (a) Incidence of aneurysms at 21 days after aneurysm induction (n = 20). (b) Rupture rate (number of mice with ruptured aneurysms/number of mice with any aneurysms) (n = 18 for the UIA-FMT+Vehicle group; n = 9 for the UIA-FMT+*H. hathewayi* group). Two-sided fisher's exact test; FMT, fecal microbiota transplantation.

Supplementary Tables

Supplementary Table 1. Baseline characteristics of the Human donors for microbiota transplantation

**Characteristics**	**Control-1**	**Control-2**	**UIA-1**	**UIA-2**
Sex	female	male	female	male
BMI, kg/m2	25.4	24.8	25.2	24.0
SBP, mmHg	134	120	139	126
DBP, mmHg	78	80	83	77
TG, mmol/L	1.16	0.91	0.96	1.32
TC, mmol/L	3.50	3.85	3.98	4.28
HDL, mmol/L	0.96	1.20	1.47	0.93
LDL, mmol/L	2.28	2.25	2.15	2.84
GLU, mmol/L	5.25	6.19	4.24	4.11

Abbreviations: BMI, body mass index; SBP, systolic blood pressure; DBP, diastolic blood pressure; TG, triglycerides; TC, total cholesterol; HDL, high-density lipoprotein; LDL, low-density lipoprotein; GLU, glucose; UIA, unruptured intracranial aneurysm.

Supplementary Table 2. Baseline characteristics of patients with UIAs and controls in the first cohort

Characteristics	Controls (n=100)	UIA patients (n=100)	*P* value
Age, y	54.7±7.1	55.4±7.6	0.302
Male sex, %	50 (50.0)	50 (50.0)	1
BMI, kg/m^2	24.8±3.5	25.8±3.8	0.055
SBP, mmHg	131.3±16.9	131.9±16.8	0.906
DBP, mmHg	83.4±10.2	82.3±10.8	0.336
TG, mmol/L	1.35 (0.98, 1.97)	1.58 (1.07, 2.18)	0.116
TC, mmol/L	5.37 (4.72, 6.01)	5.07 (4.38, 5.97)	0.075
HDL, mmol/L	1.25 (1.13, 1.53)	1.27 (1.09, 1.51)	0.666
LDL, mmol/L	2.82 (2.40, 3.30)	2.88 (2.40, 3.40)	0.568
GLU, mmol/L	5.20 (4.87, 5.60)	5.08 (4.71, 5.64)	0.489
Cigarette smoking, %			0.871
Nonsmoker	75 (75.0)	74 (74.0)	
Smoker	25 (25.0)	26 (26.0)	
Alcohol intake, %			0.487
Nondrinker	81 (81.0)	77 (77.0)	
Drinker	19 (19.0)	23 (23.0)	

Categorical variables are expressed as frequency (percent). Continuous variables are expressed as the mean ± SD or as the median (interquartile range). Abbreviations: BMI, body mass index; SBP, systolic blood pressure; DBP, diastolic blood pressure; TG, triglycerides; TC, total cholesterol; HDL, high-density lipoprotein; LDL, low-density lipoprotein; GLU, glucose; UIA, unruptured intracranial aneurysm. Statistical comparisons for percentages were performed using χ^2 analysis. Comparisons between means or medians were performed using Student's t test or the Mann-Whitney U test with the exact method.

Supplementary Table 3. Baseline characteristics of patients with UIAs and controls in the second cohort

Characteristics	Controls (n=40)	UIA patients (n=40)	*P* value
Age, y	55.3±8.6	54.6±8.5	0.491
Male sex, %	20 (50.0)	20 (50.0)	1
BMI, kg/m^2	24.5±2.3	25.3±2.3	0.133
SBP, mmHg	127.4±11.6	130.0±11.4	0.082
DBP, mmHg	80.5±6.4	83.1±9.0	0.148
TG, mmol/L	1.24 (0.96, 1.67)	1.31 (0.97, 1.76)	0.768
TC, mmol/L	4.59 (4.05, 5.68)	4.58 (3.89, 5.00)	0.090
HDL, mmol/L	1.17 (1.02, 1.55)	1.24 (1.10, 1.37)	0.970
LDL, mmol/L	2.79 (2.27, 3.83)	2.77 (2.18, 3.17)	0.254
GLU, mmol/L	5.20 (4.88, 5.69)	4.93 (4.34, 5.61)	0.202
Cigarette smoking, %			1
Nonsmoker	32 (75.0)	32 (75.0)	
Smoker	8 (25.0)	8 (25.0)	
Alcohol intake, %			0.531
Nondrinker	35 (87.5)	33 (82.5)	
Drinker	5 (12.5)	7 (17.5)	

Categorical variables are expressed as the frequency (percent). Continuous variables are expressed as the mean ± SD or as the median (interquartile range). Abbreviations: BMI, body mass index; SBP, systolic blood pressure; DBP, diastolic blood pressure; TG, triglycerides; TC, total cholesterol; HDL, high-density lipoprotein; LDL, low-density lipoprotein; GLU, glucose; UIA, unruptured intracranial aneurysm. Statistical comparisons for percentages were performed using χ^2 analysis. Comparisons between means or medians were performed using a Student's t test or the Mann-Whitney U test with the exact method.

Supplementary Table 4. Gene symbol and sequence of each primer and probe used in real-time PCR

Gene Symbol	Sequence
Col1a1	Forward: 5’-GCTCCTCTTAGGGGCCACT-3’<br>Reverse: 5’-CCACGTCTCACCATTGGGG-3’
Col2a1	Forward: 5’-CAGGATGCCCGAAAATTAGGG-3’<br>Reverse: 5’-ACCACGATCACCTCTGGGT-3’
Col3a1	Forward: 5’-CTGTAACATGGAAACTGGGGAAA-3’<br>Reverse: 5’-CCATAGCTGAACTGAAAACCACC-3’
Col4a1	Forward: 5’-CTGGCACAAAAGGGACGAG-3’<br>Reverse: 5’-ACGTGGCCGAGAATTTCACC-3’
Col4a2	Forward: 5’-GACCGAGTGCGGTTCAAAG-3’<br>Reverse: 5’-CGCAGGGCACATCCAACTT-3’
Col4a3	Forward: 5’-CAAAGGCATCAGGGGAATAACT-3’<br>Reverse: 5’-ATCCGTTGCATCCTGGTAAAC-3’
Col5a1	Forward: 5’-CTTCGCCGCTACTCCTGTTC-3’<br>Reverse: 5’-CCCTGAGGGCAAATTGTGAAAA-3’
Lama1	Forward: 5’-CAGCGCCAATGCTACCTGT-3’<br>Reverse: 5’-GGATTCGTACTGTTACCGTCACA-3’
Lama2	Forward: 5’-ATGGCTTCCCGTACTTCAGTT-3’<br>Reverse: 5’-ACCGCCAACAAAAACAGGGT-3’
Lama3	Forward: 5’-ACACCTGGGACGTGGATTG-3’<br>Reverse: 5’-CTTGCAGGGTGAATGCTTCAT-3’

Lamb1	Forward:5’- AGACCCGAAGAAAAGACAGGC -3’<br>Reverse:5’- CCATAGGGCTAGGACACCAAA -3’
Lamb2	Forward: 5’- CCCCGTCCTTGGATGTACCT -3’<br>Reverse: 5’- CAGTAGGGTTGAGGGCTATGC -3’
Lamc1	Forward: 5’- TGCCGGAGTTTGTTAATGCC -3’<br>Reverse: 5’- TGGTTGTTGTAGTCGGTCAGG -3’
Lamc2	Forward: 5’- TTGCCTCAACTGCAATGACAA -3’<br>Reverse: 5’- TCTCGATGTCGGTAAAACCCC -3’
GAPDH	Forward: 5’- CTCATGACCACAGTCCATGC -3’<br>Reverse: 5’- CACATTGGGGGTAGGAACAC -3’
Total bacteria	Forward: 5’-ACTCCTACGGGAGGCAGCAG-3’<br>Reverse: 5’- ATTACCGCGGCTGCTGG-3’
Hungatella hathewayi	Forward: 5’- CTTGACATCCCACTGAAAACAC-3’<br>Reverse: 5’- AGAGTGCCCGACTCTACTC-3’
