# Supplementary material for: Alterations of gut microbiota contribute to the progression of unruptured intracranial aneurysms
Source: Nat Commun. 2020 Jun 25;11:3218. doi: 10.1038/s41467-020-16990-3 (PMC7316982; doi:10.1038/s41467-020-16990-3)
Supplement: Supplementary file 3 — Description of Additional Supplementary Files [file 41467_2020_16990_MOESM3_ESM.docx]

**Description of Additional Supplementary Files**

**File Name: Supplementary Data 1**

**Description:** The metagenomic sequencing data of 63 samples in the first cohort, which are available from our previous study and are used in the present study (The information for each sample are shown in the table).

**File Name: Supplementary Data 2**

**Description:** Data production of 137 samples of controls and UIA patients in the first cohort.

**File Name: Supplementary Data 3**

**Description:** Data production of 80 samples of controls and UIA patients in the second cohort.

**File Name: Supplementary Data 4**

**Description:** Differentially enriched genera between controls and UIA patients in the first cohort.

**File Name: Supplementary Data 5**

**Description:** Differentially enriched species between controls and UIA patients in the first cohort.

**File Name: Supplementary Data 6**

**Description:** Differentially enriched species between controls and UIA patients in the second cohort.

**File Name: Supplementary Data 7**

**Description:** Detailed information of 220 MLGs that differed significantly in abundance between the UIA and control samples.

**File Name: Supplementary Data 8**

**Description:** Detailed information of 61 MLGs to distinguish between individuals with and without UIAs.

**File Name: Supplementary Data 9**

**Description:** Metabolic pathways that are differentially abundant between UIA patients and controls by using HUMAnN2.

**File Name: Supplementary Data 10**

**Description:** Metabolic pathways that are differentially abundant between UIA patients and controls by using KEGG orthology database.

**File Name: Supplementary Data 11**

**Description:** All identified fatty acids and amino acids and the relevant identifying information. Metabolic profiles in the sera of a subset of 60 participants (30 UIA patients and 30 controls) from the first cohort were explored by using targeted metabolomics analysis.

**File Name: Supplementary Data 12**

**Description:** Relative abundance profile at the genus level in control donors and UIA donors.

**File Name: Supplementary Data 13**

**Description:** Relative abundance profile at the genus level in mice before and after fecal transplantation.

**File Name: Supplementary Data 14**

**Description:** Differentially abundant species between mice treated with UIA patient feces and those treated with control feces.

**File Name: Supplementary Data 15**

**Description:** Detailed information of the differentially expressed genes in different groups. We performed whole-transcriptomic analysis of cerebral vessels using RNA-seq. In mice transplanted with feces from UIA patients, a total of 1212 genes (850 upregulated and 362 downregulated) were differentially expressed between the sham group and the angiotensin II- and elastase-treated group.

**File Name: Supplementary Data 16**

**Description:** The overlapping differentially expressed genes of UIAs from different species conditions. We investigated the intersection between the differentially-expressed genes (see Supplementary Data 15) and another human transcriptome-wide characterization of gene expression associated with UIA (GSE26969) and found considerable overlap between DEGs across species.

**File Name: Supplementary Data 17**

**Description:** All identified fatty acids and amino acids and the relevant identifying information. Metabolic profiles in the sera of mice transplanted with feces from UIA patients or controls were explored by using targeted metabolomics analysis.
