# Supplementary material for: Alterations of gut microbiota contribute to the progression of unruptured intracranial aneurysms
Source: Nat Commun. 2020 Jun 25;11:3218. doi: 10.1038/s41467-020-16990-3 (PMC7316982; doi:10.1038/s41467-020-16990-3)
Supplement: Supplementary file 21 — Reporting Summary [file 41467_2020_16990_MOESM21_ESM.pdf]

nature research

Corresponding author(s): Jingzhou Chen

Last updated by author(s): May 30, 2020

Reporting Summary

Nature Research wishes to improve the reproducibility of the work that we publish. This form provides structure for consistency and transparency in reporting. For further information on Nature Research policies, see Authors & Referees and the Editorial Policy Checklist .

Statistics

For all statistical analyses, confirm that the following items are present in the figure legend, table legend, main text, or Methods section.

n/a	Confirmed	
☐	☒	The exact sample size (*n*) for each experimental group/condition, given as a discrete number and unit of measurement
☐	☒	A statement on whether measurements were taken from distinct samples or whether the same sample was measured repeatedly
☐	☒	The statistical test(s) used AND whether they are one- or two-sided<br>*Only common tests should be described solely by name; describe more complex techniques in the Methods section.*
☐	☒	A description of all covariates tested
☒	☐	A description of any assumptions or corrections, such as tests of normality and adjustment for multiple comparisons
☐	☒	A full description of the statistical parameters including central tendency (e.g. means) or other basic estimates (e.g. regression coefficient) AND variation (e.g. standard deviation) or associated estimates of uncertainty (e.g. confidence intervals)
☐	☒	For null hypothesis testing, the test statistic (e.g. *F*, *t*, *r*) with confidence intervals, effect sizes, degrees of freedom and *P* value noted<br>*Give P values as exact values whenever suitable.*
☒	☐	For Bayesian analysis, information on the choice of priors and Markov chain Monte Carlo settings
☒	☐	For hierarchical and complex designs, identification of the appropriate level for tests and full reporting of outcomes
☒	☐	Estimates of effect sizes (e.g. Cohen's *d*, Pearson's *r*), indicating how they were calculated

Our web collection on statistics for biologists contains articles on many of the points above.

Software and code

Policy information about availability of computer code

Data collection	No software was used.
Data analysis	QIIME (v1.7.0); R (v3.2.1); Python (v2.7.9); Interproscan (v5.8-49.0); KOBAS (v2.0); SourceTracker (v2.0.1); MetaGeneMark (v2.10); DIAMOND (v0.7.9.58); ImageJ (v1.51); HUMAnN (2.0)

For manuscripts utilizing custom algorithms or software that are central to the research but not yet described in published literature, software must be made available to editors/reviewers. We strongly encourage code deposition in a community repository (e.g. GitHub). See the Nature Research guidelines for submitting code & software for further information.

Data

Policy information about availability of data

All manuscripts must include a data availability statement. This statement should provide the following information, where applicable:

- Accession codes, unique identifiers, or web links for publicly available datasets
- A list of figures that have associated raw data
- A description of any restrictions on data availability

All relevant data are available from the authors. The RNA sequencing data and the metagenomics data have been deposited in National Genomics Data Center, Beijing Institute of Genomics (BIG), Chinese Academy of Sciences, under accession number PRJCA001337. Furthermore, the metagenomic sequencing data from the 63 fecal samples from the controls in the first cohort have been previously published (IDs for each sample are shown in Supplementary Data 1), and are available through the EMBL European Nucleotide Archive (ENA) under BioProject accession code PRJEB13870. Functional profiling of abundance in microbial pathways was performed using KEGG orthology database, CAZy database, eggNOG database and Uniref50 database. Targeted metabolomics analysis are provided in files named Supplementary Data 11 and 17. The source data underlying Figs 1b, c, e, f, 2, 4a, 5d, 6d-h, 8a, b, f-j and Supplementary Figs 3, 7, 8b, 11, 12, 13, 15, 17a are provided as a Source Data file.

Field-specific reporting

Please select the one below that is the best fit for your research. If you are not sure, read the appropriate sections before making your selection.

☒ Life sciences ☐ Behavioural & social sciences ☐ Ecological, evolutionary & environmental sciences

For a reference copy of the document with all sections, see nature.com/documents/nr-reporting-summary-flat.pdf

Life sciences study design

All studies must disclose on these points even when the disclosure is negative.

Sample size	For clinical part, the original sample size for UIA patients and their paired controls were not determined by specific statistical hypothesis. However, from the post-hoc power analysis aspect, the 100 pairs testing and 40 pairs validation subjects could be interpreted as follow. The main purpose of the case-control studies was to detect and confirm the potential changes on gut microbiota. By this reason, with a 2-sided 0.05 alpha level, 100 pairs of subjects would offer >80% power to detect a 0.4 standardized difference between IUAs patients and the controls. Among validation set, the sample size was 40:40 (for IUA and control respectively). This number of subject also had around 80% power to demonstrate a minimum 0.6 standardized difference between the paired groups under the 2-sided 0.05 significant level (confirm the observed difference from training set). Multiplicity adjustment was not integrated into above calculations, because the findings on microbiota alternations would be validated by further animal experiments.<br>For animal study, separate interventional studies had been done regarding to different evaluation objectives. The original size of each animal study was determined by feasibility and the experience of experts in relevant research area. For example, Shikata, F. et al. Potential Influences of Gut Microbiota on the Formation of Intracranial Aneurysm. Hypertension 73, 491-496 (2019).
Data exclusions	No data were excluded from the analyses.
Replication	In human study, we performed a metagenome-wide association study in a cohort of Chinese individuals that included 100 patients with UIA and 100 controls. A cohort of 40 UIA patients and 40 controls was studied for further validation. In mouse study, all experiments were performed independently at least five times using biologically independent replicates, the number of replicates are mentioned in the figure legends section. All replication attempts were successful.
Randomization	In human study, individuals who met the following criteria were considered eligible for the study: patients diagnosed with UIAs confirmed by either digital subtraction angiography or magnetic resonance angiography and who underwent microsurgical clipping. The exclusion criteria were the following: a family history of intracranial aneurysm, ongoing infectious diseases, inflammatory bowel diseases, irritable bowel syndrome, autoimmune diseases, liver diseases, renal diseases, cancer, and diarrhea, and the use of antibiotics or probiotics within 2 months of sample collection. The samples from UIA patients were collected prior to the microsurgical clipping. Patients who were treated for their UIAs before sample collection were also excluded. Moreover, controls were included from outpatients with minor illnesses and matched to UIA patients with respect to age, sex and blood pressure. Controls were free of aneurysmal symptoms or a history of subarachnoid hemorrhage following the same exclusion criteria as used for UIA patients.<br>In mouse study, animals were randomly allocated into experimental groups.
Blinding	The investigators were blinded to group allocation during data collection and analysis.

Behavioural & social sciences study design

All studies must disclose on these points even when the disclosure is negative.

Study description	
Research sample	
Sampling strategy	
Data collection	
Timing	
Data exclusions	
Non-participation	
Randomization	

Ecological, evolutionary & environmental sciences study design

All studies must disclose on these points even when the disclosure is negative.

Study description	
Research sample	
Sampling strategy	
Data collection	
Timing and spatial scale	
Data exclusions	
Reproducibility	
Randomization	
Blinding	

Did the study involve field work? ☐ Yes ☐ No

Field work, collection and transport

Field conditions	
Location	
Access and import/export	
Disturbance	

Reporting for specific materials, systems and methods

We require information from authors about some types of materials, experimental systems and methods used in many studies. Here, indicate whether each material, system or method listed is relevant to your study. If you are not sure if a list item applies to your research, read the appropriate section before selecting a response.

Materials & experimental systems

n/a	Involved in the study	
☐	☒	Antibodies
☒	☐	Eukaryotic cell lines
☒	☐	Palaeontology
☐	☒	Animals and other organisms
☐	☒	Human research participants
☒	☐	Clinical data

Methods

n/a	Involved in the study	
☒	☐	ChIP-seq
☒	☐	Flow cytometry
☒	☐	MRI-based neuroimaging

Antibodies

Antibodies used	Goat anti-CD31 (1:400, AF3628, R&D Systems Inc., MN, USA); α-smooth muscle-Cy3™ antibody (1:1000, α-SMA, clone 1A4, C6198, Sigma-Aldrich, USA); rat anti-Ly6G (1:200, 551459, BD Pharmingen, USA); anti-F4/80 (1:200, ab6640, Abcam, MA, USA); Alexa Fluor 594-conjugated donkey anti-goat IgG (1:1000, ab150132, Abcam, MA, USA); Alexa Fluor 594-conjugated donkey anti-rat IgG (1:1000, ab150156, Abcam, MA, USA); Alexa Fluor 488-conjugated donkey anti-rat IgG (1:1000, ab150153, Abcam, MA, USA); Alexa Fluor 488-conjugated donkey anti-rabbit IgG (1:1000, ab150073, Abcam, MA, USA); Alexa Fluor 488-conjugated donkey anti-goat IgG (1:1000, ab150129, Abcam, MA, USA).
Validation	The antibodies are from commercial sources. For validation, the following methods were used: 1) use of isotype controls for analysis, 2) results from previous publications from our lab, 3) manufacturer provided validation on the same species, relevant information on the antibodies are available on the manufacture's websites.<br>Validation details of the primary antibodies are available on the manufacturers' websites:<br>anti-CD31 (1:200, AF3628, R&D Systems), https://www.rndsystems.com/cn/products/mouse-rat-cd31-pecam-1-

antibody_af3628;
α-smooth muscle-Cy3™ antibody (1:1000, α-SMA, clone 1A4, C6198, Sigma-Aldrich)
https://www.sigmaaldrich.com/catalog/product/sigma/c6198?lang=zh&region=CN
anti-Ly6G (1:200, 551459, BD Pharmingen),
https://www.bdbiosciences.com/cn/reagents/research/antibodies-buffers/immunol ogyreagents/anti-mouse-antibodies/cell-surface-antigens/purified-rat-anti-mouse-ly-6g-1a8/p/551459;
rat anti-F4/80 (1:200, ab6640, Abcam), https://www.abcam.com/f480-antibody-cia3-1-macrophage-marker-ab6640.html;
Alexa Fluor 594-conjugated donkey anti-goat IgG (1:1000, ab150132, Abcam);
https://www.abcam.cn/donkey-goat-igg-hl-alexa-fluor-594-ab150132.html
Alexa Fluor 594-conjugated donkey anti-rat IgG (1:1000, ab150156, Abcam);
https://www.abcam.cn/donkey-rat-igg-hl-alexa-fluor-594-preadsorbed-ab150156.html
Alexa Fluor 488-conjugated donkey anti-rat IgG (1:1000, ab150153, Abcam);
https://www.abcam.cn/donkey-rat-igg-hl-alexa-fluor-488-preadsorbed-ab150153.html
Alexa Fluor 488-conjugated donkey anti-rabbit IgG (1:1000, ab150073, Abcam);
https://www.abcam.cn/donkey-rabbit-igg-hl-alexa-fluor-488-ab150073.html
Alexa Fluor 488-conjugated donkey anti-goat IgG (1:1000, ab150129, Abcam)
https://www.abcam.cn/donkey-goat-igg-hl-alexa-fluor-488-ab150129.html

Eukaryotic cell lines

Policy information about cell lines

Cell line source(s)

Authentication

Mycoplasma contamination

Commonly misidentified lines
(See ICLAC register)

Palaeontology

Specimen provenance

Specimen deposition

Dating methods

☐ Tick this box to confirm that the raw and calibrated dates are available in the paper or in Supplementary Information.

Animals and other organisms

Policy information about studies involving animals; ARRIVE guidelines recommended for reporting animal research

Laboratory animals: Male wild-type C57BL/6N mice, 10 weeks old, were purchased from the National Resource Center of Model Mice. Female mice were not used to avoid any influence of sex steroids. All mice were housed in a specific pathogen-free environment (temperature, 22خ ë 2خ ; humidity, 55% ± 5%; 12-hour light/ 12-hour dark cycle) and fed a rodent diet ad libitum.

Wild animals: No wild animals were used in the study.

Field-collected samples: No field collected samples were used.

Ethics oversight: Animal experiments were approved by the Committee on the Ethics of Animal Experiments of Fuwai Hospital and complied with the National Institutes of Health's Guide for the Care and Use of Laboratory Animals, and the manuscript adheres to the Animal Research: Reporting of In Vivo Experiments (ARRIVE) guidelines.

Note that full information on the approval of the study protocol must also be provided in the manuscript.

Human research participants

Policy information about studies involving human research participants

Population characteristics: A total of 140 UIA patients and 140 age-, sex- and blood pressure-matched controls were collected from 2 cohorts. In the first cohort, individuals enrolled between April 2016 and November 2016 formed the discovery phase. A total of 100 UIA patients were consecutively enrolled from among those admitted to Beijing Tiantan Hospital. Thirty-seven controls were consecutively enrolled from Cangzhou Central Hospital, and another 63 controls were enrolled from a cohort who received biennial medical examinations at Kailuan General Hospital. Note that the metagenomic sequencing data from the 63 fecal samples from the latter controls were available from our previous study and were used in the present study. In the second cohort, individuals enrolled

	between July 2016 and December 2017 constituted the validation phase. Forty UIA patients were consecutively enrolled from among those admitted to Chinese PLA General Hospital and Beijing Tiantan Hospital. Forty controls were enrolled from Cangzhou Central Hospital and Tsinghua University Hospital. Characteristics for the individuals included in two cohorts can be found in Supplementary Tables 2 and 3.
Recruitment	Participants were recruited through the recruitment notice with informed consent. Written informed consent was provided by all study participants or their legal proxies. Individuals who met the following criteria were considered eligible for the study: patients diagnosed with UIAs confirmed by either digital subtraction angiography or magnetic resonance angiography and who underwent microsurgical clipping. The exclusion criteria were the following: a family history of intracranial aneurysm, ongoing infectious diseases, inflammatory bowel diseases, irritable bowel syndrome, autoimmune diseases, liver diseases, renal diseases, cancer, and diarrhea, and the use of antibiotics or probiotics within 2 months of sample collection. The samples from UIA patients were collected prior to the microsurgical clipping. Patients who were treated for their UIAs before sample collection were also excluded. Moreover, controls were included from outpatients with minor illnesses and matched to UIA patients with respect to age, sex and blood pressure. Controls were free of aneurysmal symptoms or a history of subarachnoid hemorrhage following the same exclusion criteria as used for UIA patients.<br>There is no self-selection bias or other biases.
Ethics oversight	The study protocol was reviewed and approved by the Human Ethics Committee, Fuwai Hospital (Approval No. 2016-732), and the study was conducted in accordance with the Principles of Good Clinical Practice and the Declaration of Helsinki. Written informed consent was given by all study participants or their legal proxies.

Note that full information on the approval of the study protocol must also be provided in the manuscript.

Clinical data

Policy information about clinical studies

All manuscripts should comply with the ICMJEguidelines for publication of clinical research and a completedCONSORT checklist must be included with all submissions.

Clinical trial registration	
Study protocol	
Data collection	
Outcomes	

ChIP-seq

Data deposition

☐ Confirm that both raw and final processed data have been deposited in a public database such as GEO.

☐ Confirm that you have deposited or provided access to graph files (e.g. BED files) for the called peaks.

Data access links<br>*May remain private before publication.*	
Files in database submission	
Genome browser session<br>(e.g. UCSC)	

Methodology

Replicates	
Sequencing depth	
Antibodies	
Peak calling parameters	
Data quality	
Software	

Flow Cytometry

Plots

Confirm that:

- ☐ The axis labels state the marker and fluorochrome used (e.g. CD4-FITC).
- ☐ The axis scales are clearly visible. Include numbers along axes only for bottom left plot of group (a 'group' is an analysis of identical markers).
- ☐ All plots are contour plots with outliers or pseudocolor plots.
- ☐ A numerical value for number of cells or percentage (with statistics) is provided.

Methodology

Sample preparation

Instrument

Software

Cell population abundance

Gating strategy

- ☐ Tick this box to confirm that a figure exemplifying the gating strategy is provided in the Supplementary Information.

Magnetic resonance imaging

Experimental design

Design type

Design specifications

Behavioral performance measures

Acquisition

Imaging type(s)

Field strength

Sequence & imaging parameters

Area of acquisition

Diffusion MRI ☐ Used ☐ Not used

Preprocessing

Preprocessing software

Normalization

Normalization template

Noise and artifact removal

Volume censoring

Statistical modeling & inference

Model type and settings

Effect(s) tested

Specify type of analysis: ☐ Whole brain ☐ ROI-based ☐ Both

Statistic type for inference
(See Eklund et al. 2016)

Correction

Models & analysis

n/a	Involved in the study
☐	☐ Functional and/or effective connectivity
☐	☐ Graph analysis
☐	☐ Multivariate modeling or predictive analysis

Functional and/or effective connectivity

Graph analysis

Multivariate modeling and predictive analysis
